# Brain-Derived Neurotrophic Factor in Acute Coronary Syndromes: Beyond Diagnosis Toward Biological Phenotyping and Risk Stratification

**DOI:** 10.3390/ijms27093826

**Published:** 2026-04-25

**Authors:** Michal Pruc, Rafal Lopucki, Katarzyna Czarnek, Şahin Çolak, Maciej Maslyk, Iwona Niewiadomska, Julia Uminska, Artur Mamcarz, Jacek Kubica, Lukasz Szarpak

**Affiliations:** 1 Institute of Medical Sciences, The John Paul II Catholic University of Lublin, 20-708 Lublin, Poland; michal.pruc@kul.pl (M.P.);; 2 Institute of Biological Sciences, The John Paul II Catholic University of Lublin, 20-708 Lublin, Poland; 3 Department of Emergency Medicine, Haydarpaşa Numune Training and Research Hospital, 34668 Istanbul, Türkiye; 4 Institute of Psychology, The John Paul II Catholic University of Lublin, 20-950 Lublin, Poland; iwona.niewiadomska@kul.pl; 5 Collegium Medicum in Bydgoszcz, Nicolaus Copernicus University, 85-067 Bydgoszcz, Poland; julia.uminska@cm.umk.pl (J.U.); jkubica@cm.umk.pl (J.K.); 6 Third Department of Internal Medicine and Cardiology, Medical University of Warsaw, 04-749 Warsaw, Poland; artur.mamcarz@wum.edu.pl; 7 Henry JN Taub Department of Emergency Medicine, Baylor College of Medicine, Houston, TX 77030, USA

**Keywords:** brain-derived neurotrophic factor, cardiac remodeling, cardiovascular biomarker, endothelial dysfunction, inflammation, phenotyping, platelet activation, thromboinflammation

## Abstract

Acute coronary syndromes (ACS) remain time-critical clinical emergencies in which early diagnosis and accurate risk stratification determine management and outcomes. Although symptoms, electrocardiography, and high-sensitivity cardiac troponin (hs-cTn) provide a reliable framework for detecting myocardial injury, they offer limited insight into plaque instability, thromboinflammatory activity, vascular repair, and post-infarction remodeling. In this narrative review, we examine the biological rationale and current clinical evidence supporting brain-derived neurotrophic factor (BDNF) as a candidate biomarker in ACS, with particular attention to pre-analytical, analytical, and phenotypic sources of heterogeneity. Available studies show that circulating BDNF concentrations vary substantially according to biological matrix, timing of sampling, ACS subtype, and assay methodology, which likely contributes to inconsistent findings across cohorts. Overall, current evidence does not support BDNF as a diagnostic alternative to hs-cTn in rule-in or rule-out pathways. However, BDNF may have value in biological phenotyping and risk stratification by reflecting platelet activation, endothelial dysfunction, inflammatory signaling, and remodeling processes after ACS. Further progress will require standardized pre-analytical procedures, separate assessment of mature BDNF and proBDNF, serial sampling, and validation in large multicenter studies.

## 1. Introduction

In patients presenting with suspected acute coronary syndromes (ACS), the immediate clinical questions are straightforward: is an acute coronary event occurring, what is the short-term risk, and who requires urgent invasive or intensive management? Contemporary algorithms from the European Society of Cardiology (ESC) and the American College of Cardiology (ACC)/American Heart Association (AHA) place high-sensitivity cardiac troponin (hs-cTn) at the center of diagnosis, alongside electrocardiography, clinical assessment, and validated risk scores such as the Global Registry of Acute Coronary Events (GRACE) and Thrombolysis In Myocardial Infarction (TIMI) scores [1,2,3]. This framework is highly effective for detecting myocardial injury and directing early treatment, but hs-cTn is fundamentally a marker of necrosis and offers little information about upstream plaque activity, platelet activation, or inflammatory tone [1,4].

Brain-derived neurotrophic factor (BDNF) has attracted interest as a candidate biomarker because it is involved in several biological pathways relevant to ACS, including endothelial signaling, platelet biology, inflammation, and neurohumoral regulation. It is produced by megakaryocytes, stored in platelets, and expressed in vascular tissue, and available data link it to plaque inflammation and instability [5,6]. At the same time, circulating BDNF is highly susceptible to methodological variability, and its diagnostic performance in ACS has been inconsistent. Although some studies have reported higher BDNF concentrations in ST-segment elevation myocardial infarction (STEMI) and associations with plaque macrophage infiltration, its value for the diagnosis of ACS remains limited [5,7]. Beyond plaque-related activity, emerging data suggest that BDNF may also reflect other layers of ACS biology, including impaired reperfusion, microvascular dysfunction, and the neuropsychiatric response to myocardial injury [8,9]. That broader scope is exactly why BDNF is unlikely to become a simple diagnostic biomarker, but may still prove useful for biological phenotyping and risk contextualization after ACS.

**Figure 1:**
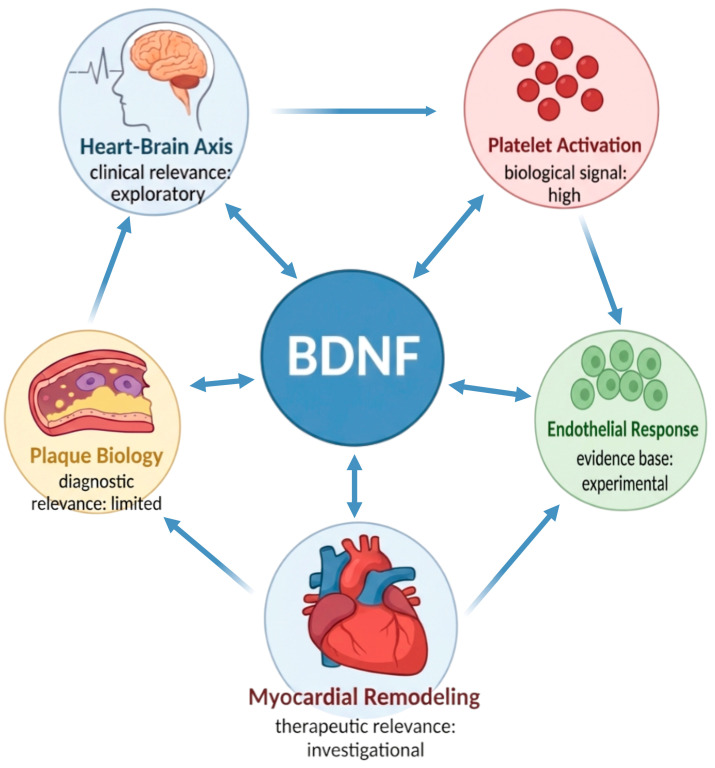
Conceptual framework of putative roles of BDNF in ACS biology. BDNF is shown as a central integrative signal potentially linked to plaque biology, platelet activation, endothelial response, myocardial remodeling, and the heart–brain axis in acute coronary syndromes (ACS). Bidirectional arrows indicate biologically plausible reciprocal interactions between BDNF and these domains, whereas peripheral arrows represent potential cross-talk between selected processes relevant to ACS pathobiology. The labels within each domain summarize the current level of clinical or biological relevance. This figure is intended as a conceptual synthesis rather than a representation of fully established causal pathways. The figure was created using Created in BioRender. Szarpak, L. (2026) https://BioRender.com/dwies28 (accessed on 15 April 2026).

The clinically relevant question, therefore, is not whether BDNF should replace established biomarkers, but whether it can refine biological phenotyping and risk assessment in selected patients with ACS. At present, the evidence supports biological plausibility rather than clinical readiness. BDNF should not be viewed as a routine diagnostic test, but as a candidate marker whose value, if confirmed, is more likely to lie in prognosis and contextual characterization of the acute event than in binary diagnosis [5,7,10]. Figure 1 provides a conceptual overview of the principal biological domains through which BDNF may be relevant to ACS, including plaque biology, platelet activation, endothelial response, myocardial remodeling, and heart-brain signaling.

## 2. Methods

This review was conceived as a focused, clinically oriented narrative review rather than a systematic review or meta-analysis. This approach was selected because the literature on BDNF in acute coronary syndromes (ACS) is biologically and methodologically heterogeneous, spanning mechanistic, translational, imaging-correlative, biomarker, and observational clinical studies. The aim was not to address a single narrowly defined question, but to provide a clinically relevant synthesis of the evidence regarding the biological plausibility of BDNF in ACS, its potential diagnostic and prognostic relevance, and its possible translational implications.

A targeted literature search was conducted in PubMed and Scopus from database inception through 2 March 2026. Search terms combined controlled vocabulary, where applicable, with free-text terms related to BDNF and ACS, including “BDNF”, “brain-derived neurotrophic factor”, “acute coronary syndrome”, “ACS”, “myocardial infarction”, “ST-segment elevation myocardial infarction”, “STEMI”, “non-ST-segment elevation myocardial infarction”, and “NSTEMI”. Reference lists of relevant original articles, review papers, and key mechanistic studies were also screened manually to identify additional publications of clinical or biological relevance. Only full-text articles published in English were considered. Conference abstracts, editorials, and letters were not used as primary sources of evidence. This approach may have introduced language and publication bias, which should be considered when interpreting the scope of the review. A detailed description of the full search strategy is presented in Supplementary Table S1.

The review was guided by predefined thematic domains addressing: (1) the biological rationale linking BDNF to ACS pathobiology; (2) the potential diagnostic significance of BDNF in ACS; (3) its role in risk stratification and prognosis after ACS; and (4) its possible translational and therapeutic relevance. Studies were selected on the basis of relevance to these domains, direct applicability to ACS biology or clinical phenotyping, and sufficient methodological detail to permit meaningful interpretation.

Priority in the synthesis was given to human studies directly addressing ACS, myocardial infarction, plaque phenotype, reperfusion or microvascular dysfunction, clinical outcomes, and related cardiovascular phenotypes relevant to acute coronary injury. Experimental and translational studies were included when they offered mechanistic insight directly relevant to cardiovascular injury, platelet biology, endothelial function, inflammation, myocardial remodeling, or heart–brain signaling. When clinical and experimental evidence addressed the same question, greater interpretive weight was given to clinical studies, particularly prospective cohorts and studies with clear phenotypic characterization, imaging or outcome correlation, standardized sampling, and clinically relevant endpoints. Studies conducted in non-cardiovascular settings were considered only when they clarified mechanisms central to the interpretation of BDNF signaling in ACS.

Because the field includes biologically non-equivalent BDNF-related measures, studies were not treated as interchangeable solely because they referred to BDNF. Separate consideration was given to total circulating BDNF, mature BDNF, proBDNF, BDNF methylation, and BDNF genotype, as these reflect related but distinct biological processes and should not be interpreted as a single analyte across studies. Because these BDNF-related measures are biologically related but analytically non-equivalent, they should not be interpreted interchangeably; their principal characteristics and reporting requirements are summarized in Table 1.

As this was a narrative rather than a systematic review, formal study-level quality scoring and quantitative risk-of-bias assessment were not performed. Instead, the literature was appraised using a domain-based narrative approach, with particular attention to study design, cohort composition, ACS phenotype, biological matrix, timing of sampling, assay methodology, sample handling, endpoint definition, and susceptibility to confounding. Particular emphasis was placed on methodological and analytical issues especially relevant to BDNF research in ACS, including serum-versus-plasma differences, platelet-derived confounding, pre-analytical variability, and the frequent failure to distinguish mature BDNF from proBDNF.

The final synthesis was organized within predefined interpretive domains, including BDNF biology and mechanisms of action, pathophysiological relevance to ACS, diagnostic performance, plaque and reperfusion phenotypes, prognosis and risk stratification, heart–brain interactions, and translational implications. Within each domain, emphasis was placed on biological plausibility, consistency of findings, methodological sources of heterogeneity, and the distinction between observations that may inform future clinical phenotyping and those that remain hypothesis-generating. The purpose of this review was not to provide an exhaustive catalogue of all published associations, but to offer a clinically grounded and methodologically explicit interpretation of the literature most relevant to BDNF in ACS.

## 3. Biology and Mechanisms of BDNF Action

BDNF is synthesized as a precursor protein rather than secreted directly in its final active form. The precursor form, proBDNF, can undergo intracellular and extracellular proteolytic cleavage to generate mature BDNF. This processing step is biologically important because proBDNF and mature BDNF are not interchangeable analytes: proBDNF preferentially activates p75 neurotrophin receptor (p75NTR)/sortilin-related signaling, whereas mature BDNF primarily signals through tropomyosin receptor kinase B (TrkB) [11,12]. Although BDNF is classically considered a neurotrophin, it is also expressed in several non-neural compartments relevant to ACS, including endothelial cells, immune cells, vascular smooth muscle cells, megakaryocytes, and platelets [12,13]. For this reason, both proteolytic processing and tissue source are central to the interpretation of circulating BDNF in cardiovascular disease [11,12,13].

Referring to BDNF as a single, biologically uniform entity is therefore an oversimplification that obscures important mechanistic differences. At least two biologically distinct forms must be considered: proBDNF and mature BDNF. These isoforms exert divergent effects through different receptor systems. ProBDNF preferentially binds the p75 neurotrophin receptor (p75NTR)/sortilin receptor complex and activates proapoptotic and proinflammatory signaling pathways, whereas mature BDNF signals primarily through tropomyosin receptor kinase B (TrkB), promoting prosurvival, trophic, and anti-inflammatory responses [11,12,14,15]. Experimental data therefore indicate that the biological effects of BDNF are not unidirectional, but depend on ligand form, receptor repertoire, tissue context, and the local balance between proteolytic processing and receptor expression.

In cellular and animal models, BDNF participates in several pathways relevant to vascular and inflammatory biology. Experimental data indicate that BDNF supports endothelial cell survival within the cardiac microvasculature and contributes to microvascular integrity [12,16]. BDNF also promotes embryonic stem cell differentiation into endothelial cells through a pathway involving microRNA-214, enhancer of zeste homolog 2 (EZH2), and endothelial nitric oxide synthase (eNOS) [17]. In cellular models, BDNF stimulates migration of young coronary endothelial cells via full-length TrkB/phosphoinositide 3-kinase (PI3K)/Akt signaling, whereas in senescent endothelial cells its effects appear to be mediated by the truncated TrkB-T1 receptor [12]. Additional experimental work has shown that BDNF can protect endothelial cells against NLR family pyrin domain containing 3 (NLRP3) inflammasome-induced pyroptosis and modulate glucose metabolism through the Kruppel-like factor 2 (KLF2)/hexokinase 1 (HK1) pathway [18]. BDNF is also closely linked to platelet biology. Megakaryocytes express the BDNF gene, and circulating platelets represent one of the principal extra-neural storage sites for the protein, with concentrations reported to be 100- to 1000-fold higher than in neurons [5,13]. Platelet release of BDNF occurs predominantly after protease-activated receptor 1 (PAR1) activation and largely involves the pool stored in alpha granules, although release is incomplete and platelets themselves express a truncated TrkB receptor lacking the tyrosine kinase domain [13]. In experimental settings, BDNF enhances platelet aggregation through Rac1, protein kinase C, and PI3K signaling and stimulates secretion of angiogenic and inflammatory mediators [13]. Interpretation of circulating BDNF is further complicated by major pre-analytical variability. Serum concentrations are substantially higher than plasma concentrations, largely because coagulation triggers platelet degranulation and BDNF release [19,20,21]. Measured levels are influenced by clotting time, centrifugation protocol, anticoagulant type, storage temperature, long-term storage, and freeze–thaw cycles [19,20,22]. Serum and plasma BDNF concentrations do not correlate closely, suggesting that they reflect different biological pools, while currently available immunoassays vary in their ability to distinguish mature BDNF from proBDNF [19,21,23,24]. Taken together, these data establish biological plausibility for a role of BDNF in vascular, inflammatory, and platelet-related pathways relevant to ACS. However, most of this evidence is mechanistic, and it should not be interpreted as direct proof of clinical usefulness in diagnosis or risk stratification.

## 4. BDNF in the Pathogenesis of ACS

BDNF is highly expressed within atherosclerotic lesions, particularly in macrophages and coronary artery smooth muscle cells, and appears to be involved in several processes central to ACS pathobiology, including plaque destabilization, inflammation, oxidative stress, and vascular repair [5,6]. Its expression is increased in both the intima and adventitia of atherosclerotic coronary arteries, with prominent localization in macrophages and smooth muscle cells. In vitro, stimulation of coronary artery smooth muscle cells with BDNF increases NADPH oxidase activity and reactive oxygen species generation, providing a mechanistic link between BDNF, oxidative stress, and plaque vulnerability [6].

BDNF levels correlate with macrophage infiltration and with the absence of healed plaques, supporting a role in plaque inflammation and destabilization. Higher circulating BDNF concentrations have been reported in patients with STEMI and in those with macrophage-rich plaques, whereas lower levels are observed in the presence of healed plaques, further suggesting an association between BDNF and acute plaque events [5]. A small but interesting extension of this concept is that BDNF may also relate to what happens after plaque disruption, namely downstream perfusion and microvascular integrity. In a recent retrospective single-center study of 100 patients with STEMI treated with percutaneous coronary intervention (PCI), patients were categorized according to post-procedural TIMI flow, with the no-reflow/slow-flow group compared against patients with normal reperfusion. In that cohort, higher circulating BDNF levels were independently associated with the no-reflow phenotype after multivariable adjustment and were accompanied by worse TIMI flow grade and lower left ventricular ejection fraction. The study also identified other expected severity-related correlates, including diabetes, higher Killip class, lower estimated glomerular filtration rate, multivessel disease, higher thrombus burden, and higher BNP. However, because the study was retrospective, single-center, and did not provide long-term follow-up data, the findings should be interpreted as hypothesis-generating rather than practice-informing [8]. By contrast, a cross-sectional angiographic study of 125 patients with suspected coronary artery disease reported that serum mature BDNF levels were significantly lower in patients with coronary slow flow than in those with normal coronary flow. Blood samples were obtained before angiography, and lower mature BDNF was independently associated with the coronary slow flow phenotype and inversely related to mean TIMI frame count. This study is important because it assessed serum mature BDNF rather than total circulating BDNF, which may partly explain why its direction of association differed from studies focused on total BDNF in STEMI. At the same time, its single-center cross-sectional design and lack of longitudinal outcomes limit mechanistic and clinical inference [25]. Taken together, these findings suggest that BDNF-related signaling may span a biologically plausible bridge between plaque instability and downstream perfusion disturbance, although the current evidence is still observational, methodologically heterogeneous, and too limited for firm mechanistic conclusions.

The precursor form, proBDNF, is upregulated in proinflammatory monocytes/macrophages after acute myocardial infarction and contributes to maintenance of a proinflammatory phenotype. At the same time, experimental evidence indicates that neutralization of proBDNF aggravates cardiac dysfunction and enlarges infarct size, at least in part through upregulation of matrix metalloproteinase-9, underscoring the complex and context-dependent role of this pathway in post-infarction remodeling and resolution of inflammation [14].

Genetic variation further modifies BDNF biology. The Val66Met polymorphism influences BDNF secretion and function, and available data suggest that it may affect post-infarction remodeling and macrophage phenotype, although its precise clinical significance in ACS remains uncertain. Taken together, these findings support the view that BDNF sits at the crossroads of plaque biology, inflammation, oxidative stress, and reparative adaptation in ACS, with its effects shaped by isoform, cellular context, and genetic background [5,6,14].

## 5. BDNF as a Diagnostic Biomarker

From the perspective of the acute cardiac care clinician, BDNF does not currently address a diagnostic gap that hs-cTn has already filled with exceptional accuracy and extensive clinical validation. Contemporary ACS guidelines from the ESC and the ACC/AHA do not assign BDNF any role in rule-in or rule-out strategies or routine biomarker-based triage [1,26,27,28]. Hs-cTn assays, particularly when applied in 0/1 h or 0/2 h algorithms, achieve sensitivities approaching 100%, negative predictive values exceeding 99%, and area under the curve values of at least 0.92 for the diagnosis of non-ST-segment elevation myocardial infarction (NSTEMI) [1,29,30]. By contrast, there are no accepted decision thresholds for BDNF, no standardized biological matrix for testing, and no validated sampling windows that would support routine use in frontline clinical decision-making [5].

This does not mean that the signal carried by BDNF is irrelevant. Much of the apparent inconsistency in the literature is better explained by differences in biological matrix, sampling time, and ACS phenotype than by genuine biological contradiction. Some early clinical studies reported lower serum BDNF levels in patients with coronary artery disease (CAD) than in controls; in one such study, serum BDNF concentrations were 30.69 ± 5.45 ng/mL in CAD compared with 46.58 ± 7.95 ng/mL in controls [10,31]. By contrast, other investigators reported higher serum BDNF concentrations in acute myocardial infarction than in stable angina (1730 vs. 877 pg/mL), with correlations to soluble P-selectin suggesting an association with platelet activation [32]. These observations are not mutually exclusive. They underscore that BDNF behaves differently in plasma and serum because of platelet degranulation during coagulation, and that both sampling time and ACS phenotype materially influence the observed signal [19,20,21].

The most clinically relevant diagnostic observation is that BDNF may reflect plaque biology more closely than myocardial necrosis itself. In the study by Montone and colleagues, which included 126 patients with ACS, serum BDNF levels were higher in STEMI than in NSTEMI [5]. In the subgroup that underwent optical coherence tomography (n = 53), BDNF independently predicted macrophage infiltration, with an odds ratio (OR) of 2.856 (95% confidence interval (CI) 1.151–7.090; *p* = 0.024), and was associated with the absence of healed plaque morphology (OR 0.438, 95% CI 0.185–0.992; *p* = 0.050) [5]. This is arguably the setting in which BDNF is biologically most informative: not as a competitor to troponin, but as a potential laboratory correlate of a fresh, inflamed, thrombosis-prone plaque phenotype. However, these signals derive from relatively small cohorts, limited imaging subgroups, and surrogate correlates of plaque activity rather than from studies designed to support clinical implementation [5,6].

Crucially, biological informativeness should not be conflated with clinical utility. Even if BDNF captures aspects of plaque activity or platelet-inflammatory signaling, no study to date has demonstrated that it meaningfully improves diagnostic discrimination, calibration, or reclassification beyond hs-cTn-based algorithms, electrocardiography, and standard clinical assessment. There are no validated data showing incremental area under the curve (AUC), net reclassification improvement (NRI), or decision-analytic benefit sufficient to support incorporation of BDNF into routine ACS diagnostic pathways [1,5,26,27,28,29,30].

There are also signals suggesting that BDNF may reflect the severity of the acute presentation. In a single-center study of 78 patients with acute myocardial infarction, higher BDNF concentrations were associated with acute heart failure (HF) and higher Killip class, and the combination of BDNF with cancer antigen 125 (CA 125) improved discrimination of patients with HF complications, with a sensitivity of 91.02% and a specificity of 81.63% [33]. BDNF concentrations also correlated positively with Killip class (r = 0.745, *p* < 0.001) [33]. These findings are of interest, but they remain far removed from practical implementation in ACS diagnosis, as they relate to a specific complication rather than to standardized diagnostic decision-making. Notably, plasma BDNF concentrations are generally lower in patients with HF and correlate inversely with B-type natriuretic peptide levels and New York Heart Association functional class, indicating that the relationship between BDNF and cardiac dysfunction is complex and may differ substantially between acute and chronic settings [34,35].

The available data suggest that BDNF may capture biologically relevant features of ACS, but they do not support its use as a diagnostic biomarker. Much of the heterogeneity across studies can be attributed to differences in biological matrix, timing of sampling, assay methodology, and clinical phenotype [5,19,20,29]. More importantly, no convincing evidence has shown that BDNF adds clinically actionable diagnostic value beyond hs-cTn, electrocardiography, and standard clinical assessment. Its more plausible role lies in contextual phenotyping of ACS rather than in binary acute diagnosis. Associations with no-reflow after PCI or with coronary slow flow do not make BDNF a diagnostic biomarker for ACS; if anything, they reinforce the view that BDNF may be more relevant to biological subphenotyping than to frontline diagnostic decision-making [8,25]. A concise overview of representative human studies evaluating BDNF-related measures across ACS and related coronary phenotypes is provided in Table 2.

## 6. BDNF in Risk Prediction and Prognosis in ACS

If BDNF holds greater promise in any domain of ACS care, it is in prognosis rather than diagnosis. The strongest data come from epigenetic studies. In patients with ACS, increased methylation of the BDNF gene has been associated with a higher risk of adverse long-term cardiovascular outcomes even after adjustment for major clinical covariates. In a prospective study of 969 patients with ACS followed for 5–12 years, higher BDNF methylation was independently associated with an increased risk of major adverse cardiac events (MACE), with a hazard ratio (HR) of 1.45 (95% CI 1.17–1.78) [36]. Higher BDNF methylation was also associated with elevated interleukin-6 levels, suggesting a link between epigenetic regulation and inflammatory burden [36]. Subsequent work showed that the adverse prognostic effect of increased BDNF methylation is amplified in the presence of comorbid depression and may be attenuated by escitalopram treatment in depressed patients with ACS [36,37]. Another useful way to read this literature is that BDNF-related risk may be strongly context dependent rather than autonomous. In particular, the prognostic effect of higher BDNF methylation appears to be modified by inflammatory burden. In one study, greater average BDNF methylation predicted worse long-term cardiovascular outcomes mainly in patients with higher IL-18 levels, suggesting that BDNF-related vulnerability may become clinically visible especially in a proinflammatory biological background rather than in isolation [38].

The genetic dimension is similarly relevant. The Val66Met polymorphism (rs6265), a functional variant within the BDNF gene, has been linked to susceptibility to unstable angina, angiographic disease burden, and long-term outcomes [39,40,41,42]. In a Chinese cohort of 1417 patients, the Met/Met genotype was associated with a lower risk of unstable angina (OR 0.53, *p* = 0.005) and lower high-sensitivity C-reactive protein levels compared with Val carriers [42]. By contrast, data from the CATHGEN cohort of 5510 White patients suggested that the Val/Val genotype was associated with more extensive coronary artery disease (OR 1.17 for a greater number of diseased vessels, 95% CI 1.06–1.30, *p* = 0.002), lower left ventricular ejection fraction, and a higher risk of death or myocardial infarction (HR 1.12, 95% CI 1.01–1.24, *p* = 0.034) compared with Met carriers [40]. Experimental evidence from knock-in mice further showed that the Met/Met genotype is associated with a depressive-like phenotype, a hypercoagulable state, platelet hyperreactivity, and increased susceptibility to arterial thrombosis, while in humans Met homozygosity has been linked to a higher risk of acute myocardial infarction [39,43].

Of particular interest in ACS is the interaction between BDNF biology and the patient’s psychosocial profile. In a study of 611 patients with ACS followed for 5–12 years, the Val66Met polymorphism modified the association between vulnerable personality traits and adverse long-term cardiac outcomes, with significant gene-environment interactions observed [41]. Specifically, the associations between vulnerable personality type, as well as the individual dimensions of agreeableness and neuroticism, and MACE were statistically significant only in the presence of the Met allele [41]. In addition, among patients with ACS and comorbid depression, carriers of the Met allele had higher remission rates with escitalopram treatment, raising the possibility that BDNF genotype may predict antidepressant response in this setting [44]. One useful refinement of the prognostic literature is that BDNF-related risk appears to be strongly context dependent rather than autonomous. In a prospective ACS cohort, higher average BDNF methylation predicted worse long-term outcomes mainly in patients with higher IL-18 levels, including composite MACE, myocardial infarction, and repeat PCI, whereas the association was much weaker in those with lower inflammatory burden [38]. This suggests that BDNF-related vulnerability may become clinically relevant particularly in a proinflammatory context rather than as an isolated biomarker signal. Although not a long-term outcome study, the reported association of higher pre-PCI BDNF with no-reflow, lower TIMI flow grade, and lower left ventricular ejection fraction in STEMI also suggests that BDNF may track early severity-related phenotypes relevant to short-term prognosis [8].

These findings should be interpreted cautiously. Much of the prognostic literature derives from single-center or regional cohorts; clinical endpoints are not uniform across studies; and some analyses combine patients with stable coronary disease, ACS, and chronic coronary syndromes under broad cardiovascular categories [7]. The direction of association has also not been entirely consistent across populations. In community-based cohorts, higher serum BDNF levels have been associated with a lower risk of future cardiovascular events and mortality. In the Framingham Heart Study, higher serum BDNF was inversely associated with cardiovascular disease risk (HR per one-standard-deviation increase 0.88, 95% CI 0.80–0.97, *p* = 0.01) and mortality (HR 0.87, 95% CI 0.80–0.93, *p* = 0.0002) [45]. Similarly, in patients with angina pectoris, low plasma BDNF was an independent predictor of 4-year major coronary events (adjusted HR 1.25, 95% CI 1.10–1.41, *p* < 0.01) and mortality (adjusted HR 1.29, 95% CI 1.11–1.47, *p* < 0.01) [46]. Such findings should not be directly extrapolated to the acute phase of ACS, in which BDNF may reflect platelet activation and acute inflammatory signaling rather than chronic protective mechanisms [5,7,32].

Taken together, current evidence suggests that BDNF-related measures may provide adjunctive prognostic information in ACS and may help refine biological risk phenotyping, particularly when interpreted alongside epigenetic, genetic, inflammatory, and psychosocial variables. What remains unproven is whether they improve discrimination, calibration, or reclassification beyond established biomarkers and validated risk models. At present, BDNF should therefore be regarded as a potentially informative adjunctive signal rather than as a proven tool for incremental prognostic modeling [36,41,45].

## 7. BDNF in the Heart-Brain Axis and Psychosocial Modifiers of Prognosis

Experimental studies suggest that BDNF participates in bidirectional signaling between myocardial injury and the central nervous system. In conditional knockout models, myocardial infarction was associated with an increase in circulating BDNF accompanied by upregulation of BDNF expression in the brain, but not in the heart, whereas ablation of afferent cardiac nerves or disruption of neuronal BDNF signaling blunted this response and worsened cardiac dysfunction [47]. Neuronal BDNF deficiency also aggravated post-infarction remodeling, resulting in more severe systolic dysfunction, greater cardiac enlargement, and increased cardiomyocyte death, while peripheral BDNF administration partially restored the phenotype [47]. Additional experimental work suggests that the temporal profile of BDNF may also be relevant, with early post-infarction increases followed by later declines during adverse remodeling, in parallel with worsening left ventricular dysfunction, adrenergic denervation, and impaired angiogenesis [48]. Taken together, these findings support the concept that BDNF may form part of a broader neurocardiac adaptive response to myocardial injury [47,48,49].

There is also relevant preclinical evidence linking post-infarction neuroinflammation and altered central BDNF signaling to depression-like behavior. In ovariectomized female rats after myocardial infarction, depression-like behavior was associated with increased proinflammatory cytokine activity in the plasma, paraventricular nucleus, and prefrontal cortex, together with reduced mature BDNF expression in the prefrontal cortex; pentoxifylline attenuated both the behavioral abnormalities and the decrease in central mature BDNF. Complementary work in male rats showed that anti-inflammatory treatment with minocycline attenuated post-infarction depression-like behavior and reduced central and peripheral inflammatory activation, supporting neuroinflammation as a plausible intermediate pathway between myocardial injury and mood-related behavioral changes. Taken together, these animal data suggest that myocardial injury may promote neuroinflammation, disrupt central BDNF-related signaling, and contribute to depressive symptoms. However, this evidence remains preclinical and should not be extrapolated directly to psychiatric diagnosis in patients with ACS [50,51].

Human data are more limited and should be interpreted primarily in a prognostic rather than psychiatric diagnostic framework. Observational studies in ACS cohorts have linked BDNF methylation and BDNF-related genetic variation with depression vulnerability, selected distress-related phenotypes, antidepressant response, and long-term cardiovascular outcomes [36,37,44,52]. Higher BDNF methylation has been associated with both depressive disorder early after ACS and persistence of depressive symptoms during follow-up, while its adverse prognostic effect appears to intersect with inflammatory activity and long-term cardiovascular risk [36,37]. Similarly, the Val66Met polymorphism has been linked to depression vulnerability and to differential response to escitalopram in patients with ACS [36,44]. From the perspective of ACS phenotyping, the main relevance of this literature is therefore not diagnostic, but prognostic: it suggests that, in a subset of patients, psychosocial phenotype may modify cardiovascular risk through mechanisms that are at least partly biologically mediated [36,53,54].

At the same time, this literature requires considerable interpretive restraint. Most human data are observational and highly vulnerable to confounding by somatic comorbidity, smoking, platelet-related effects, antidepressant treatment, sex, and age. In patients with coronary heart disease, apparent associations between lower BDNF and depressive symptoms weakened after adjustment for these factors, while heart failure itself remained associated with lower BDNF levels [55]. BDNF should therefore not be interpreted as a specific marker of depression in ACS, but rather as a biologically mixed signal that may contribute to integrated psychocardiologic and prognostic phenotyping. For now, this axis remains biologically compelling but exploratory, and its direct relevance to routine ACS management is still uncertain [36,37,55].

## 8. Translational and Therapeutic Perspectives

If BDNF is to acquire translational relevance in ACS, it is unlikely to do so as an isolated biomarker. A more realistic translational scenario would be an integrated framework combining BDNF-related measures with markers of myocardial injury, inflammatory burden, plaque phenotype, vascular or microvascular dysfunction, and psychosocial assessment [5,9,38]. This may be especially relevant across the transition from the acute ACS phase to early recovery, where reperfusion quality, remodeling, anxiety, depression, and rehabilitation participation begin to interact in ways that are not captured by hs-cTn alone. The rationale for such approaches is well established in cardiovascular medicine, where combinations of biomarkers have consistently outperformed single-marker strategies in risk stratification [3,56]. For BDNF, however, meaningful translation will require substantially greater methodological rigor than is currently available. Future studies will need serial rather than single time-point sampling, a clearly defined biological matrix, rigorous pre-analytical standardization, and separate quantification of mature BDNF and proBDNF [19,20,22]. At present, plasma appears methodologically preferable to serum when the aim is to minimize platelet-derived confounding, although this should be viewed as a working analytical preference rather than a definitively established standard [19,20,22]. Integration with imaging, genotyping, epigenetic profiling, and established clinical risk models will also be necessary to determine whether BDNF adds information beyond existing biomarkers and conventional phenotyping [5,26,36,57].

In experimental models, several signals suggest that modulation of the BDNF-TrkB axis may influence post-infarction remodeling and myocardial recovery. Preclinical studies have shown that TrkB agonists such as LM22A-4 and 7,8-dihydroxyflavone can attenuate adverse post-infarction changes, including left ventricular dysfunction, adrenergic denervation, and impaired angiogenesis [48,58]. Additional experimental work from rat post-infarction models indicates that exercise training modulates the BDNF-TrkB pathway, preventing reductions in mature BDNF, phosphorylated calcium/calmodulin-dependent protein kinase II (CaMKII), and phosphorylated Akt in the myocardium, while β-adrenergic signaling may also influence myocardial BDNF content. These data derive from controlled animal experiments rather than human ACS studies and should therefore be interpreted as preclinical mechanistic evidence, not as clinical proof of efficacy [48,49,59,60]. These observations are biologically and translationally interesting, but they derive from controlled preclinical settings with defined timing, dosing, and model-specific conditions. These interventions have not been validated in clinical ACS care, and they do not yet support clinical targeting of the BDNF axis in ACS [48,49,58,59,60].

The current clinical reality remains considerably more limited. There are no standardized assays suitable for routine implementation, no validated decision thresholds, no consensus regarding the optimal biological matrix or sampling window, and no prospective interventional trials showing that BDNF-guided strategies improve outcomes in ACS. Moreover, the field remains constrained by substantial analytical heterogeneity, variable cohort design, and an incomplete separation of mechanistic, prognostic, and therapeutic questions [14,15,19,20,22,26,39,41,44,61]. At present, BDNF is best regarded as a biologically plausible research marker that may help refine future multimodal phenotyping strategies, rather than as a tool ready for clinical decision-making. A concise summary of the current diagnostic, prognostic, and translational relevance of BDNF in ACS is provided in Table 3. At present, these observations should be regarded as hypothesis-generating rather than practice-informing. No BDNF-based strategy is currently ready for clinical implementation in ACS.

## 9. Limitations

This review has several limitations. First, it is a narrative rather than a systematic review, and some degree of selection bias is therefore unavoidable. Although the aim was to provide a clinically focused and mechanistically integrated appraisal of the field, the literature was not synthesized within a formal quantitative framework. Second, the available evidence on BDNF in ACS is highly heterogeneous at both the biological and methodological levels. Studies differ substantially with respect to biological matrix, timing of sampling, pre-analytical handling, storage conditions, assay platform, and the extent to which mature BDNF and proBDNF are distinguished. These differences are not minor technical details; they materially influence measured values and likely account for a substantial part of the apparent inconsistency across studies. Third, much of the clinical literature derives from relatively small, observational, and often single-center cohorts with heterogeneous populations and endpoints. In many reports, patients with STEMI, NSTEMI, stable coronary disease, and broader cardiovascular phenotypes are analyzed within overlapping frameworks, which limits both comparability and generalizability. In addition, adjustment for important confounders, including inflammatory burden, psychiatric comorbidity, medication use, and renal or metabolic status, is not uniform across studies. Finally, an important part of the biological rationale for BDNF comes from experimental and translational work, which provides mechanistic plausibility but cannot be directly extrapolated to clinical decision-making in ACS. Another problem is that studies grouped under the label of BDNF research often do not examine the same biological or clinical construct. Some assess total circulating BDNF, others mature BDNF, others methylation or genotype, and they do so across very different phenotypes ranging from ACS and STEMI to post-infarction rehabilitation, chronic coronary disease, HF, no-reflow, or coronary slow flow. This makes cross-study comparison harder than it may appear at first glance and creates an easy path toward overinterpretation, especially in the psychosocial literature. The single most important limitation of the field remains the absence of analytical standardization sufficient to allow meaningful comparison across studies or translation into practice. Until this problem is addressed, the interpretation of BDNF in ACS will remain constrained by methodological uncertainty, and its clinical applicability will remain largely investigational.

## 10. Conclusions

At present, BDNF is not a biomarker ready for routine diagnostic use in ACS. It does not compete with hs-cTn, lacks standardized analytical conditions and decision thresholds, and remains highly sensitive to biological matrix, timing of sampling, and clinical context. Its more plausible relevance lies in capturing the biological context of ACS rather than in supporting binary diagnosis.

The strongest rationale for continued investigation lies in prognosis and biological phenotyping. Current data suggest that BDNF-related measures may help identify patients with greater thrombo-inflammatory activity, reperfusion or microvascular vulnerability, or a more adverse heart–brain response to myocardial injury, but this potential remains investigational. BDNF is not another troponin; it is a compartment- and phase-dependent signal whose clinical relevance will depend on analytical standardization and prospective validation against established biomarkers and risk models.

Among BDNF-related analytes, no form is currently ready for clinical use. If one analyte is prioritized for future translational research, analytically well-characterized mature BDNF probably has the clearest near-term potential, because it maps more directly onto biologically interpretable TrkB-related signaling and already has some early human phenotype-level data in coronary disease. By contrast, proBDNF is mechanistically compelling, particularly in inflammatory and monocyte/macrophage biology, but remains further from clinical implementation because human ACS data are still sparse and assay standardization is limited.

## Figures and Tables

**Table 1:** Analytical non-equivalence of BDNF-related measures in ACS research.

Measure	What It Reflects Most Directly	Main Analytical Problem	Minimum Reporting Requirement
Serum total BDNF	Circulating signal strongly influenced by platelet release during clotting	Highly sensitive to clotting time and pre-analytical handling; may overrepresent platelet-derived BDNF rather than free circulating levels	Serum matrix, clotting duration, centrifugation protocol, storage conditions, assay used
Plasma total BDNF	Lower-concentration circulating pool less shaped by clotting-related platelet release	Sensitive to anticoagulant choice, processing delay, centrifugation protocol, and low assay range	Plasma matrix, anticoagulant, processing time, centrifugation protocol, assay characteristics
Mature BDNF	TrkB-related trophic and potentially reparative signaling	Often not analytically separated from proBDNF, despite distinct biological effects	Assay specificity, confirmation of mature/proBDNF discrimination, biological matrix, timing of sampling
proBDNF	p75NTR/sortilin-related proapoptotic and proinflammatory signaling	Biologically distinct from mature BDNF but often discussed under the same umbrella term	Explicit proBDNF assay, matrix, sampling context, analytical validation
BDNF methylation	Epigenetic regulation of BDNF-related vulnerability	Does not reflect circulating protein concentration and may be influenced by inflammation, psychiatric comorbidity, and treatment exposure	Tissue/source of DNA, locus or CpG region assessed, platform, adjustment for major covariates
BDNF genotype (Val66Met)	Inherited variation affecting intracellular trafficking, secretion, and biological susceptibility	Static genetic marker rather than a dynamic biomarker of the acute ACS phase	Variant tested, genotyping method, ancestry/ethnicity, phenotype definition, model adjustment
Tissue or plaque BDNF expression	Local vascular, plaque, or myocardial biological activity	Mechanistically informative but not directly equivalent to blood-based measurement	Tissue source, phenotype/context, detection method, relation to circulating measurements if available

Footnote: BDNF-related measures used in ACS research are biologically related but analytically non-equivalent. They should not be interpreted interchangeably under the single label of “BDNF” without explicit consideration of analyte type, biological matrix, timing of sampling, assay characteristics, and pre-analytical handling. Abbreviations: ACS, acute coronary syndromes; BDNF, brain-derived neurotrophic factor; CpG, cytosine-phosphate-guanine dinucleotide; DNA, deoxyribonucleic acid; p75NTR, p75 neurotrophin receptor; proBDNF, precursor brain-derived neurotrophic factor; TrkB, tropomyosin receptor kinase B.

**Table 2:** Representative human studies evaluating BDNF-related measures across ACS and related coronary phenotypes.

Study	Population	Matrix/Analyte	Sampling Context	Main Finding	Potential Clinical Relevance	Key Limitation
Montone et al. 2021 [5]	126 patients with ACS (55 STEMI, 71 NSTEMI); OCT subgroup n = 53	Serum BDNF (isoform-unspecified)	ACS presentation	Higher BDNF in STEMI than NSTEMI; associated with macrophage infiltration and absence of healed plaque	Plaque-inflammatory phenotyping rather than binary diagnosis	Small cohort; limited OCT subgroup; surrogate plaque correlates
Zhang et al. 2025 [8]	100 patients with STEMI treated with PCI	BDNF (isoform-unspecified)	Acute STEMI hospitalization with post-PCI TIMI flow stratification	Higher BDNF associated with no-reflow, worse TIMI flow, and lower LVEF	Reperfusion and severity phenotyping after STEMI	Retrospective single-center cohort; no long-term follow-up
Zhang et al. 2025 [25]	125 patients with suspected CAD (77 CSFP, 48 normal flow)	Serum mature BDNF	Before coronary angiography	Lower mature BDNF associated with coronary slow flow and higher TIMI frame count	Microvascular/flow-related phenotyping rather than ACS diagnosis	Cross-sectional single-center study; not an ACS-only cohort
Lorgis et al. 2010 [32]	40 patients undergoing coronary angiography: 20 with acute myocardial infarction and 20 with stable angina pectoris	Serum BDNF	Acute MI versus stable angina comparison	Higher serum BDNF in MI than in stable angina; in MI, BDNF correlated with soluble P-selectin and showed a non-significant trend with soluble CD40 ligand	Platelet-activation/thrombo-inflammatory phenotyping in acute MI rather than routine diagnosis	Very small study; not an undifferentiated ACS diagnostic cohort; no validated thresholds or outcome validation
Wu et al. 2019 [33]	78 patients with AMI	Serum BDNF plus CA125	Acute AMI	Higher BDNF associated with acute HF and higher Killip class; combined model improved discrimination of HF complications	Early complication/severity stratification in acute MI	Small single-center study; relates to complications rather than ACS diagnosis itself

Footnote: Human studies evaluating BDNF-related measures in ACS and related coronary phenotypes are biologically related but clinically and analytically non-equivalent. They should not be interpreted interchangeably under the single label of BDNF without explicit consideration of analyte type, biological matrix, sampling context, assay characteristics, and the clinical phenotype under study. Abbreviations: ACS, acute coronary syndromes; AMI, acute myocardial infarction; BDNF, brain-derived neurotrophic factor; CAD, coronary artery disease; CA125, cancer antigen 125; CSFP, coronary slow flow phenomenon; HF, heart failure; LVEF, left ventricular ejection fraction; MI, myocardial infarction; NSTEMI, non-ST-segment elevation myocardial infarction; OCT, optical coherence tomography; PCI, percutaneous coronary intervention; STEMI, ST-segment elevation myocardial infarction; TIMI, Thrombolysis In Myocardial Infarction.

**Table 3:** Current clinical interpretation of BDNF in acute coronary syndromes.

Domain	Best Evidence	Main Limitation	Current Clinical Readiness
Diagnosis	Human association studies suggest that BDNF reflects platelet-inflammatory activation and plaque-related activity more closely than myocardial necrosis.	No standardized assay, no validated thresholds, major dependence on matrix, timing, and pre-analytical handling; studies are small and heterogeneous.	Not suitable for routine diagnosis. No role in rule-in/rule-out pathways for ACS.
Plaque biology/inflammatory phenotyping	OCT-based and observational studies link higher BDNF levels with macrophage-rich plaques and absence of healed plaque features.	Evidence is limited to small cohorts, restricted imaging subgroups, and surrogate correlates of plaque activity.	Potential research marker of plaque phenotype, but not ready for clinical use.
Microvascular/reperfusion phenotype	Small observational studies suggest associations between circulating BDNF and impaired reperfusion after STEMI, including no-reflow after PCI, while serum mature BDNF has also been inversely associated with coronary slow flow and TIMI frame count.	Evidence is limited to small, heterogeneous, largely single-center cohorts using different biological readouts (total BDNF versus mature BDNF) and different clinical phenotypes.	Hypothesis-generating only. Potentially relevant for mechanistic phenotyping, but not for routine clinical use.
Prognosis and risk stratification	The most consistent human signals involve BDNF methylation, Val66Met, and their interaction with inflammation, depression, and long-term outcomes.	Evidence is largely observational, analytically heterogeneous, and insufficiently validated; incremental value beyond established biomarkers and risk scores remains unproven.	Promising for prognostic research, but not for independent clinical decision-making.
Heart-brain axis/psychosocial modifiers	Experimental studies support a neurocardiac adaptive response after myocardial infarction; observational studies link BDNF-related variation with depression vulnerability, suicidal ideation, antidepressant response, and long-term risk after ACS.	Mechanistic evidence is mainly preclinical, whereas human psychiatric data remain observational and are highly vulnerable to confounding by somatic comorbidity, smoking, platelet-related effects, and treatment exposure.	Biologically compelling but exploratory. Relevant mainly to integrated psychocardiologic and prognostic phenotyping, not to psychiatric diagnosis or routine ACS management.
Therapeutic relevance	Preclinical studies suggest that TrkB agonists, exercise-related signaling, and β-adrenergic pathways may influence BDNF-associated remodeling.	No validated BDNF-based intervention, no prospective ACS trials, and no standardized therapeutic framework.	No current therapeutic role in ACS. BDNF-targeted strategies remain investigational.

Footnote: Clinical readiness reflects current applicability to routine ACS care rather than biological plausibility. Abbreviations: ACS—acute coronary syndromes; BDNF—brain-derived neurotrophic factor; OCT—optical coherence tomography; STEMI—ST-segment elevation myocardial infarction; PCI—percutaneous coronary intervention.

## Data Availability

No new data were created in this study. Data sharing is not applicable to this article. This article is a narrative review based on previously published literature identified through searches of PubMed and Scopus and manual screening of reference lists. All sources supporting the conclusions of this review are cited in the reference list.

## Supplementary Materials

The following supporting information can be downloaded at: https://www.mdpi.com/article/10.3390/ijms27093826/s1, Supplementary File S1: Full Literature Search Strategy.

## Abbreviations

The following abbreviations are used in this manuscript:

ACC	American College of Cardiology
ACS	Acute Coronary Syndromes
AHA	American Heart Association
AUC	Area Under the Curve
BDNF	Brain-Derived Neurotrophic Factor
CA 125	Cancer Antigen 125
CAD	Coronary Artery Disease
CaMKII	Calcium/Calmodulin-Dependent Protein Kinase II
CI	Confidence Interval
CpG	Cytosine-Phosphate-Guanine
DNA	Deoxyribonucleic Acid
eNOS	Endothelial Nitric Oxide Synthase
ESC	European Society of Cardiology
EZH2	Enhancer of Zeste Homolog 2
GRACE	Global Registry of Acute Coronary Events
HF	Heart Failure
HK1	Hexokinase 1
HR	Hazard Ratio
hs-cTn	High-Sensitivity Cardiac Troponin
IL-18	Interleukin-18
IL-6	Interleukin-6
KLF2	Kruppel-Like Factor 2
MACE	Major Adverse Cardiac Events
NADPH	Nicotinamide Adenine Dinucleotide Phosphate
NLRP3	NLR Family Pyrin Domain Containing 3
NRI	Net Reclassification Improvement
NSTEMI	Non-ST-Segment Elevation Myocardial Infarction
OCT	Optical Coherence Tomography
OR	Odds Ratio
p75NTR	p75 Neurotrophin Receptor
PAR1	Protease-Activated Receptor 1
PCI	Percutaneous Coronary Intervention
PI3K	Phosphoinositide 3-Kinase
proBDNF	Precursor Brain-Derived Neurotrophic Factor
STEMI	ST-Segment Elevation Myocardial Infarction
TIMI	Thrombolysis In Myocardial Infarction
TrkB	Tropomyosin Receptor Kinase B
Val66Met	Valine-to-Methionine Substitution at Codon 66 of BDNF
