# Supplementary material for: Brain-Derived Neurotrophic Factor in Acute Coronary Syndromes: Beyond Diagnosis Toward Biological Phenotyping and Risk Stratification
Source: Int J Mol Sci. 2026 Apr 25;27(9):3826. doi: 10.3390/ijms27093826 (PMC13163832; doi:10.3390/ijms27093826)
Supplement: Supplementary file 1 [file ijms-27-03826-s001.zip › ijms-4241010-supplementary.pdf]

# Supplementary File S1
## Full Literature Search Strategy

## Exact PubMed search strings

### Core broad search

```
(
 "Brain-Derived Neurotrophic Factor"[Mesh]
 OR BDNF[Title/Abstract]
 OR "brain-derived neurotrophic factor"[Title/Abstract]
 OR "brain derived neurotrophic factor"[Title/Abstract]
 OR proBDNF[Title/Abstract]
 OR "pro-BDNF"[Title/Abstract]
 OR "mature BDNF"[Title/Abstract]
 OR "BDNF methylation"[Title/Abstract]
 OR "BDNF gene methylation"[Title/Abstract]
 OR "BDNF polymorphism"[Title/Abstract]
 OR "BDNF Val66Met"[Title/Abstract]
 OR Val66Met[Title/Abstract]
 OR rs6265[Title/Abstract]
)
AND
(
 "Acute Coronary Syndrome"[Mesh]
 OR "Myocardial Infarction"[Mesh]
 OR "Coronary Thrombosis"[Mesh]
 OR "acute coronary syndrome"[Title/Abstract]
 OR "acute coronary syndromes"[Title/Abstract]
 OR ACS[Title/Abstract]
 OR "myocardial infarction"[Title/Abstract]
 OR "acute myocardial infarction"[Title/Abstract]
 OR AMI[Title/Abstract]
 OR STEMI[Title/Abstract]
 OR NSTEMI[Title/Abstract]
 OR "ST-segment elevation myocardial infarction"[Title/Abstract]
 OR "ST segment elevation myocardial infarction"[Title/Abstract]
 OR "ST-elevation myocardial infarction"[Title/Abstract]
 OR "non-ST-segment elevation myocardial infarction"[Title/Abstract]
 OR "non-ST segment elevation myocardial infarction"[Title/Abstract]
 OR "non-ST-elevation myocardial infarction"[Title/Abstract]
 OR "non-ST elevation myocardial infarction"[Title/Abstract]
 OR "non-ST-elevation acute coronary syndrome"[Title/Abstract]
 OR "non-ST elevation acute coronary syndrome"[Title/Abstract]
 OR "NSTE-ACS"[Title/Abstract]
 OR "NSTE ACS"[Title/Abstract]
 OR "unstable angina"[Title/Abstract]
 OR "coronary thrombosis"[Title/Abstract]
)
```

### Targeted supplementary search: plaque phenotype / imaging correlates

```
(
 BDNF[Title/Abstract]
 OR "brain-derived neurotrophic factor"[Title/Abstract]
 OR "brain derived neurotrophic factor"[Title/Abstract]
 OR proBDNF[Title/Abstract]
 OR "mature BDNF"[Title/Abstract]
)
AND
(
 "acute coronary syndrome"[Title/Abstract]
 OR "myocardial infarction"[Title/Abstract]
 OR STEMI[Title/Abstract]
 OR NSTEMI[Title/Abstract]
 OR "unstable angina"[Title/Abstract]
```

)
AND
(
 plaque[Title/Abstract]
 OR vulnerability[Title/Abstract]
 OR "plaque instability"[Title/Abstract]
 OR macrophage*[Title/Abstract]
 OR "optical coherence tomography"[Title/Abstract]
 OR OCT[Title/Abstract]
)

## Targeted supplementary search: reperfusion / microvascular phenotype

(
 BDNF[Title/Abstract]
 OR "brain-derived neurotrophic factor"[Title/Abstract]
 OR "brain derived neurotrophic factor"[Title/Abstract]
 OR proBDNF[Title/Abstract]
 OR "mature BDNF"[Title/Abstract]
)
AND
(
 "acute coronary syndrome"[Title/Abstract]
 OR "myocardial infarction"[Title/Abstract]
 OR STEMI[Title/Abstract]
 OR NSTEMI[Title/Abstract]
 OR "unstable angina"[Title/Abstract]
)
AND
(
 "no-reflow"[Title/Abstract]
 OR "no reflow"[Title/Abstract]
 OR reperfusion[Title/Abstract]
 OR "microvascular obstruction"[Title/Abstract]
 OR "microvascular dysfunction"[Title/Abstract]
 OR "coronary slow flow"[Title/Abstract]
 OR "TIMI frame count"[Title/Abstract]
)

## Targeted supplementary search: epigenetic / genetic modifiers

(
 BDNF[Title/Abstract]
 OR "brain-derived neurotrophic factor"[Title/Abstract]
 OR "brain derived neurotrophic factor"[Title/Abstract]
 OR proBDNF[Title/Abstract]
 OR "mature BDNF"[Title/Abstract]
 OR Val66Met[Title/Abstract]
 OR rs6265[Title/Abstract]
)
AND
(
 "acute coronary syndrome"[Title/Abstract]
 OR "myocardial infarction"[Title/Abstract]
 OR STEMI[Title/Abstract]
 OR NSTEMI[Title/Abstract]
 OR "unstable angina"[Title/Abstract]
)
AND
(
 methylat*[Title/Abstract]
 OR epigen*[Title/Abstract]
 OR genotype[Title/Abstract]
 OR polymorphism*[Title/Abstract]
 OR variant*[Title/Abstract]
 OR Val66Met[Title/Abstract]
 OR rs6265[Title/Abstract]

```
)
```

### Targeted supplementary search: heart-brain / psychosocial modifiers

```
(
  BDNF[Title/Abstract]
  OR "brain-derived neurotrophic factor"[Title/Abstract]
  OR "brain derived neurotrophic factor"[Title/Abstract]
  OR proBDNF[Title/Abstract]
  OR "mature BDNF"[Title/Abstract]
  OR Val66Met[Title/Abstract]
  OR rs6265[Title/Abstract]
)
AND
(
  "acute coronary syndrome"[Title/Abstract]
  OR "myocardial infarction"[Title/Abstract]
  OR STEMI[Title/Abstract]
  OR NSTEMI[Title/Abstract]
  OR "unstable angina"[Title/Abstract]
)
AND
(
  depress*[Title/Abstract]
  OR anxiety[Title/Abstract]
  OR psychosocial[Title/Abstract]
  OR personality[Title/Abstract]
  OR "heart-brain"[Title/Abstract]
  OR neurocardi*[Title/Abstract]
  OR antidepress*[Title/Abstract]
)
```

## Exact Scopus search strings

### Core broad search

```
TITLE-ABS-KEY(
  BDNF
  OR "brain-derived neurotrophic factor"
  OR "brain derived neurotrophic factor"
  OR proBDNF
  OR "pro-BDNF"
  OR "mature BDNF"
  OR "BDNF methylation"
  OR "BDNF gene methylation"
  OR "BDNF polymorphism"
  OR "BDNF Val66Met"
  OR Val66Met
  OR rs6265
)
AND
TITLE-ABS-KEY(
  "acute coronary syndrome"
  OR "acute coronary syndromes"
  OR ACS
  OR "myocardial infarction"
  OR "acute myocardial infarction"
  OR AMI
  OR STEMI
  OR NSTEMI
  OR "ST-segment elevation myocardial infarction"
  OR "ST segment elevation myocardial infarction"
  OR "ST-elevation myocardial infarction"
  OR "non-ST-segment elevation myocardial infarction"
  OR "non-ST segment elevation myocardial infarction"
  OR "non-ST-elevation myocardial infarction"
```

```
  OR "non-ST elevation myocardial infarction"
  OR "non-ST-elevation acute coronary syndrome"
  OR "non-ST elevation acute coronary syndrome"
  OR "NSTE-ACS"
  OR "NSTE ACS"
  OR "unstable angina"
  OR "coronary thrombosis"
)
```

## Targeted supplementary search: plaque phenotype / imaging correlates

```
TITLE-ABS-KEY(
  BDNF
  OR "brain-derived neurotrophic factor"
  OR "brain derived neurotrophic factor"
  OR proBDNF
  OR "mature BDNF"
)
AND
TITLE-ABS-KEY(
  "acute coronary syndrome"
  OR "myocardial infarction"
  OR STEMI
  OR NSTEMI
  OR "unstable angina"
)
AND
TITLE-ABS-KEY(
  plaque
  OR vulnerability
  OR "plaque instability"
  OR macrophage*
  OR "optical coherence tomography"
  OR OCT
)
```

## Targeted supplementary search: reperfusion / microvascular phenotype

```
TITLE-ABS-KEY(
  BDNF
  OR "brain-derived neurotrophic factor"
  OR "brain derived neurotrophic factor"
  OR proBDNF
  OR "mature BDNF"
)
AND
TITLE-ABS-KEY(
  "acute coronary syndrome"
  OR "myocardial infarction"
  OR STEMI
  OR NSTEMI
  OR "unstable angina"
)
AND
TITLE-ABS-KEY(
  "no-reflow"
  OR "no reflow"
  OR reperfusion
  OR "microvascular obstruction"
  OR "microvascular dysfunction"
  OR "coronary slow flow"
  OR "TIMI frame count"
)
```

## Targeted supplementary search: epigenetic / genetic modifiers

```
TITLE-ABS-KEY(
  BDNF
```

```
  OR "brain-derived neurotrophic factor"
  OR "brain derived neurotrophic factor"
  OR proBDNF
  OR "mature BDNF"
  OR Val66Met
  OR rs6265
)
AND
TITLE-ABS-KEY(
  "acute coronary syndrome"
  OR "myocardial infarction"
  OR STEMI
  OR NSTEMI
  OR "unstable angina"
)
AND
TITLE-ABS-KEY(
  methylat*
  OR epigen*
  OR genotype
  OR polymorphism*
  OR variant*
  OR Val66Met
  OR rs6265
)
```

## Targeted supplementary search: heart-brain / psychosocial modifiers

```
TITLE-ABS-KEY(
  BDNF
  OR "brain-derived neurotrophic factor"
  OR "brain derived neurotrophic factor"
  OR proBDNF
  OR "mature BDNF"
  OR Val66Met
  OR rs6265
)
AND
TITLE-ABS-KEY(
  "acute coronary syndrome"
  OR "myocardial infarction"
  OR STEMI
  OR NSTEMI
  OR "unstable angina"
)
AND
TITLE-ABS-KEY(
  depress*
  OR anxiety
  OR psychosocial
  OR personality
  OR "heart-brain"
  OR neurocardi*
  OR antidepress*
)
```
